# Hirsute or Hairless? Two Proteins May Spell the Difference

**DOI:** 10.1371/journal.pbio.0030035

**Published:** 2004-12-28

If you're a cat fancier, you're well aware that hair follicles are expendable. The product of a spontaneous mutation that caught a cat breeder's eye, *le chat nu*, would quickly succumb in the wild—its winter coat consists of little more than a ridge of fur down the midback and tail—and needs special care to thrive as a pet. Hairless animals in the lab, on the other hand, can be very instructive. Understanding how hair develops sheds light on the fundamental processes that generate a wide range of tissues and organs, including the lungs, cornea, and mammary glands.

How complex, three-dimensional structures emerge from single sheets of cells is a fundamental question in developmental biology. The dispensability of hair follicles makes them the perfect model system for studying this question—specifically, how structures and organs develop from buds. In a new study, Elaine Fuchs and colleagues use a three-pronged approach—involving gene expression analysis, transgenic mice, and cell cultures—to study how epithelial buds, the precursors of hair follicles, form. Their experiments point to two key actors in a signaling pathway that molds a targeted cluster of cells into a hair bud.

During the budding process, overlapping signaling pathways from two adjacent embryonic cell layers—the epithelium and the mesenchyme—direct morphogenesis. The mesenchymal cells initiate the cell-to-cell “crosstalk” that controls bud formation by first directing a small cluster of epithelial cells to form a placode, the pouch that forms hair plugs. The placode in turn directs underlying mesenchymal cells to form the base of the hair follicle, called the dermal papilla, and both structures contribute to the mature hair follicle. During development, cells are constantly bombarded with external signals. The trick is figuring out which signals trigger the transcriptional and behavioral properties in cells that spur bud formation.

In previous experiments, Fuchs and colleagues showed that reducing expression of E-cadherin—a membrane protein that forms the adhesive junctions between epidermal cells—is essential for allowing the cell remodeling required for bud formation. Here, the authors analyze the timing of external signals against the response of targeted cells to determine how targeted cells translate signals into changes in cell adhesion and remodeling, proliferation, and differentiation—the agents of most types of organogenesis.[Fig pbio-0030035-g001]

**Figure pbio-0030035-g001:**
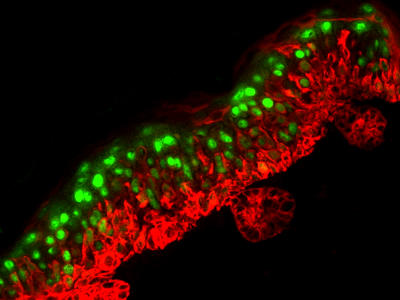
Transgenic epidermis expressing Snail (red) results in expanded keratin 1 expression (green)

Since Snail, a protein that impedes the transcription of a subset of genes, functions in many developmental processes requiring epithelial remodeling, the authors reasoned it might do the same in hair bud formation. Working with developing mouse embryos, they saw a spike in Snail expression on embryonic day 17.5, coinciding with hair bud formation, enhanced cell proliferation, and the down-regulation of E-cadherin. Artificially sustaining Snail expression in the skin of transgenic mice caused abnormal levels of cell proliferation in the epidermis and reduced cell adhesion.

Working with skin keratinocytes, precursors of hair fibers, Fuchs and colleagues explored several signaling proteins known to be involved in bud formation as possible activators of Snail expression. When the authors treated keratinocytes with small amounts of one stimulator, TGF-β2, they saw “rapid and transient induction of Snail.” Snail proteins were absent from 17.5-day-old knockout mice lacking TGF-β2 but not from their nonmutant littermates. Conversely, transgenic mice with elevated TGF-β2 signaling activity displayed ectopic expression of Snail. Knockout mice lacking TGF-β2 also showed higher levels of E-cadherin—normally down-regulated by Snail—than their nonmutant littermates.

Altogether, these findings suggest that TGF-β2 signaling transiently induces Snail, which in turn down-regulates E-cadherin and activates a proliferation pathway in the developing bud. Reduced E-cadherin, the authors conclude, appears to contribute to Snail-mediated enhanced proliferation by allowing proteins normally sequestered at the membrane to operate in a proliferation pathway after the number of cellular junctions diminishes. By identifying which molecules are active in specific cell types at specific developmental stages, this study lays the foundation for dissecting the mechanisms that connect two key processes—intercellular remodeling and proliferation—in epithelial development. And since the consequences of TGF-β2 activity seen here in the hair bud more closely resemble certain types of skin cancer progression than skin development, a mechanistic understanding of hair follicle development promises to shed light on how skin cancer develops as well.

